# Associations of mobile phone addiction with suicide ideation and suicide attempt: findings from six universities in China

**DOI:** 10.3389/fpubh.2023.1338045

**Published:** 2024-01-19

**Authors:** Wenhua Wang, Mingyang Wu, Zhongliang Zhu, Le Ma, Lei Zhang, Hui Li

**Affiliations:** ^1^Department of Neonatology, The First Affiliated Hospital of Xi'an Jiaotong University, Xi'an, China; ^2^Shaanxi Provincial Health Industry Association Service Center, Xi'an, China; ^3^Department of Maternal and Child Health, Xiangya School of Public Health, Central South University, Changsha, China; ^4^Institute of Maternal and Infant health, Medical college of Northwest University, Xi'an, China; ^5^School of Public Health, Xi'an Jiaotong University Health Science Center, Xi'an, China; ^6^Shaanxi Medical Association, Xi'an, China

**Keywords:** mobile phone addiction, suicide ideation, suicide attempt, epidemiology, college students

## Abstract

**Background:**

Mobile phones are becoming indispensable for life and have changed various aspects of people's lives. The psychological impacts of excessive mobile phone use have emerged as an impressive problem among college students. However, little is known about the associations of mobile phone addiction with suicide ideation and suicide attempt.

**Methods:**

A cross-sectional study was conducted with students from six universities in 2022. We collected the socio-demographic characteristics, suicide ideation, suicide attempt, psychosocial factors (depressive symptoms, social support, sleep quality), and health-related characteristics (smoking, drinking, body mass index). Mobile phone addiction was ascertained by the Mobile Phone Addiction Tendency Scale (MPATS). The associations of mobile phone addiction with suicide ideation and suicide attempt were estimated using binary logistic regression and restricted cubic splines regression.

**Results:**

A total of 18,723 college students [6,531 males (34.9%) and 12,192 females (65.1%)] were included in the final analysis. Eleven percent of participants had a history of suicide ideation, and 1.8% of participants had engaged in suicide attempt. A total of 5,553 students (29.7%) met the criteria of mobile phone addiction (MPATS score ≥48), and the average score on the MPATS was 39.5 ± 13.0. After adjustment for potential covariates, mobile phone addiction was significantly associated with increased odds of suicide ideation (OR, 1.70; 95% CI, 1.53–1.88) and suicide attempt (OR, 1.48; 95% CI, 1.18–1.86). Gender did not affect the associations of mobile phone addiction with suicide ideation and suicide attempt (*P for interaction* > 0.05). The restricted cubic splines regression displayed a nonlinear dose-response association between MPATS score and risk of suicide ideation (*P for non-linearity* < 0.001), while a monotonically increasing risk of suicide attempt was found to be associated with an increasing MPATS score (*P for non-linearity* = 0.420).

**Conclusions:**

Mobile phone addiction is associated with suicide ideation and suicide attempt among college students. The findings indicate that early examination, prevention, and intervention for mobile phone addiction may benefit the prevent and control of suicide.

## 1 Introduction

Approximately one million people around the world died by suicide every year (1). In both China and Japan, suicide is the leading cause of death among youth aged 15–34, resulting in substantial loss of life (2). Suicide was regarded as a continuous process, which usually involves suicide ideation, suicide plan, suicide attempt, and completed suicide (3). Recent global surveys indicated that 10%−20% of adolescents report having had suicide ideation or suicide attempt during the last year (4). More specifically, approximately 25 attempts occur for every suicide death, and an even greater number of people consider suicide (5, 6). Over the past two decades, there has been a considerable increase in a variety of studies on suicide among youth (7), but limited progress has been made in reducing suicide rates (8, 9). Suicide among youth remains a heavy burden on the state and society. Therefore, further exploring the influencing factors of suicide is essential for decreasing the incidence of suicide behavior and, ultimately, reducing suicidal death.

There is an increasing interest in revealing the adverse health effects of excessive mobile phones use among youth, especially for the generation born after 1995, who were tagged as iGens by Twenge (10). The iGens have spent their entire adolescence in the era of mobile phones, their social interactions and mental health might be shaped by the combined influence of mobile phones and social media. For the above reasons and circumstances, mobile phone addiction has become the major problem among the iGens, which is similar to the internet addiction with the characteristics of withdrawal symptoms, tolerance, loss of control, and adverse effects (11). Many previous epidemiological surveys found a high rate of mobile phone addiction among college students. For example, Zhang et al. (12) found that the prevalence of problematic mobile phone use among Chinese university students was as high as 26.1%. As a systematic review reported, China was at the highest incidence of mobile phone addiction (13). Furthermore, the COVID-19 outbreak has brought many impacts on people's lives, particularly on current college students who are the first generation of iGens. The quarantine measures for COVID-19 forced college students to take online classes at home or in dormitories, which increased their use of mobile phones and resulted in an increased risk of mobile phone addiction (14, 15). Several studies indicated that individuals who engage in mobile phone addiction may suffer many adverse consequences, such as bodily and headache pain, dry eyes, depression, anxiety, sleep disorders, and poor academic performance (16–20). Among the various health effects, the psychological impacts of mobile phone addiction are of the greatest concern, but the relationship between mobile phone addiction and suicide is still unclear.

Given the amount of time spent on mobile phones, iGens with mobile phone addiction or prolonged use of mobile phones will increase the risk of exposure to suicide pictures, videos, texts, and cases. What's more, mobile phones also provide an important way for iGens to find and join suicide peers, where they can share their experiences of suicide and even be encouraged (21). In addition, studies have shown that over-indulgence in various apps on mobile phones can decrease the interest in face-to-face relationships (22), resulting in non-adaptation to real life, may causing a series of problems (e.g., depression, anxiety, sleep quality) (18), which will lead to suicide in turn. Mobile phone use has also been linked to structural and functional abnormalities in brain areas related to cognitive control and emotional regulation. Zou et al. (23) found that decreased integrity of brain white matter is associated with mobile phone addiction, and lower white matter integrity is believed to be associated with suicide in adolescents (24). Therefore, it is natural to hypothesize that mobile phone addiction is likely to increase the risk of suicide.

To date, a few studies have revealed the relationships of mobile phone addiction with suicide ideation and suicide attempt. However, the findings from these studies are not consistent. Shinetsetseg et al. (25) conducted a web-based survey of 54,948 middle and high school students in Korea, suggested that mobile phone addiction leads to significant higher risk of suicide ideation and suicide attempt. Similarly, findings from 1,609 senior high school students in China also indicated a significant relationship between mobile phone addiction and suicide ideation or suicide attempt (26). In addition to high school students, there were several studies on college students. Wan Ismail et al. (27) showed that mobile phone addiction was positively related to suicide in 525 college students from six public universities in Malaysian. Recently, Hu et al. (28) also found significant association of mobile phone addiction with suicide ideation among 1,042 college students at a Chinese university. However, a recent study of 439 college students found no relationship between suicide ideation and mobile phone addiction (29). While, most previous studies on the association between mobile phone addiction and suicide among college students have been conducted at only one university, from specific majors, or only from public universities, and all with a small sample size. Adding to the limited relevant studies at present, further research is required.

Therefore, this study aims to examine the associations of mobile phone addiction with suicide ideation and suicide attempt using a large sample of college students from six universities.

## 2 Methods

### 2.1 Study design and participants

This cross-sectional survey was conducted among undergraduates across six representative universities in Shaanxi Province, which is located in northwest China, from October to November 2022. In short, we used multi-stage random cluster sampling to choose participants. First, with assistance from the educational bureaus, we sampled six of 57 universities in Shaanxi province, including four of 34 public universities and two of 23 private universities. Then, we randomly selected two to four classes from all faculties and grades at each sampled university, inviting a total of 20,165 undergraduates from 559 selected classes to join the study. We excluded students refused to participate, submitted in a short time (<500 s), and those invalid questionnaire assessed by logic questions. In the end, 18,723 students were included in the final analysis, leading to a response rate of 95.4% (18,723/20,165).

Trained investigators conducted the survey and they were available to explain to students any questions they may have about the structured questionnaire. Before the survey, we conducted a two-stage training to inform students of the purpose and procedures in detail. First, two class officers from each selected class were assembled for a 27 min standardized training with the participation of school leaders. Subsequently, the class officers conducted another training to all students in each selected classes, and instructed other classmates to complete the questionnaire. All participants finished the questionnaire with an average time of 27 min. Ethics approval was obtained from The Second Affiliated Hospital of Xi'an Jiaotong University (Approval number: 2022-248).

### 2.2 Measures

#### 2.2.1 Socio-demographic characteristics

Socio-demographic information, including gender, grade, race, registered permanent residence (rural, urban), siblings (yes, no), and parental educational attainment (middle school or under, high school, college or above), were collected.

#### 2.2.2 Mobile phone addiction

Mobile Phone Addiction Tendency Scale (MPATS) developed by Xiong et al. (30) for Chinese college students was used to measure mobile phone addiction. It included 16 items with four dimensions, including salience, withdrawal symptoms, social comfort, and mood changes. The items were rated from 1 (very inconsistent) to 5 (very consistent), with a total score ranging from 16 to 80. A higher total score indicates a higher level of mobile phone addiction. A score of 48 or more is considered to be mobile phone addiction. In this study, Cronbach α coefficient was 0.94.

#### 2.2.3 Suicide ideation and suicide attempt

Suicide ideation was assessed by the question “During the last week, what was your level of desire to actively attempt suicide.” “During the last week, to what extent do you want external forces to end your life.” A “weak” or “moderate to strong” response to the preceding two questions implies the presence of suicide ideation (31). Suicide attempt was measured by “During the last year, did you ever attempt suicide?” An affirmative answer to this question was defined as suicide attempt. In the present study, the Cronbach α coefficient was 0.83.

#### 2.2.4 Depression symptoms

Depression symptoms were measured by using the Self-Rating Depression Scale (SDS), which is developed by Zung (32). It included 20 questions that evaluates 10 positive symptoms and 10 negative symptoms for nearly 1 week. All items were scored from 1 (no or little time) to 4 (majority or all of the time). A higher score indicates more severe depression symptoms. The presence of depression symptoms was defined as a standard score higher than 50. The Cronbach α was 0.88 in this study.

#### 2.2.5 Social support

Adolescent Social Support Scale was used to assess social support (33). It consists of 16 items with three dimensions (subjective support, objective support and support utilization). The items were scored from 1 (inconsistent) to 5 (consistent), with higher scores indicating greater level of perceived social support. The Cronbach α was 0.98 in this study.

#### 2.2.6 Sleep disorders

Pittsburgh Sleep Quality Index (PSQI) developed by Buysse (34) was used to measure sleep disorders. It contains 19 items to evaluates the sleep quality during the last month. The total score of seven domains produced a global PSQI score ranging from 0 to 21, with a score of 8 or more defined as sleep disorders. The Cronbach α was 0.85 in this study.

#### 2.2.7 Health-related characteristics

We also asked health-related characteristics information include smoking, drinking, height, and weight. Current smokers were participants who have smoked one or more cigarettes in the past 30 days. Current alcohol drinkers were defined as those who had consumed at least one glass of wine during the past 30 days. Body mass index (BMI) was calculated by dividing body weight (kg) by the square of height (m).

### 2.3 Statistical analysis

Continuous and categorical variables were presented as mean (SD) and frequencies, respectively. χ^2^ test or *t-*test was performed to compare the distributions of the different characteristics.

Four sets of binary logistic regression were constructed to estimate the odds ratios (ORs) and 95% confidence interval (CIs) of mobile phone addiction with suicide ideation and suicide attempt separately, with adjustment for potential confounders ascertained based on prior publications (28, 29). Model 1 is a crude model, unadjusted for other factors. In model 2, we adjusted for gender, grade, and race. In model 3, we also adjusted for siblings, registered permanent residence, parental educational attainment, smoking, drinking, and BMI. In model 4, we additionally adjusted for depressive symptoms, social support, and sleep disorders.

Given that the gender-specific differences in the prevalence of suicidal behaviors were reported in previous study (35). We additionally performed subgroup analyses to examine any gender differences in the associations of mobile phone addiction with suicide ideation and suicide attempt. We inserted gender × mobile phone addition as an interaction term in the regression models to obtain *P* value for interaction. In addition, restricted cubic splines regression with three knots at 10th, 50th, and 90th percentiles of MPATS score were used to estimate the dose-response relationship of the MPATS score with the odds of suicide ideation and suicide attempt.

R 4.0.2 software (https://www.r-project.org/) was used for all data analyses.

## 3 Results

Table 1 presents the baseline characteristics of the participants. Among 18,723 participants, the majority were Han nationality (97.0%), 65.9% were females, 29.5% were from one-child families, and 54.0% were rural residents. Overall, 2020 participants (10.8%) had a history of suicide ideation, and 362 participants (1.9%) had engaged in suicide attempt. We also observed that 5,553 participants (29.7%) met the criteria of mobile phone addiction (MPATS score ≥48), and the average MPATS score of all participants was 39.5 ± 13.0. The average scores for the four dimensions of withdrawal symptoms, salience, social comfort, and mood changes were 16.0 ± 5.4, 8.7 ± 3.4, 7.6 ± 3.0, and 7.1 ± 2.8, respectively.

**Table 1:** Characteristics of participants.

**Characteristics**	**Suicide ideation**		***P*-value**	**Suicide attempt**		***P*-value**
	**Never**	**Ever**		**Never**	**Ever**	
**Gender**, ***n*** **(%)**						
Male	5,862 (35.1)	669 (33.1)	0.083	6,415 (34.9)	116 (32)	0.276
Female	10,841 (64.9)	1,351 (66.9)		11,946 (65.1)	246 (68)	
**Grade**, ***n*** **(%)**						
1st	4,884 (29.2)	569 (28.2)	< 0.001	5,336 (29.1)	117 (32.3)	0.068
2nd	3,950 (23.6)	518 (25.6)		4,371 (23.8)	97 (26.8)	
3rd	3,872 (23.2)	527 (26.1)		4,317 (23.5)	82 (22.7)	
4th+	3,997 (23.9)	406 (20.1)		4,337 (23.6)	66 (18.2)	
**Race**, ***n*** **(%)**						
Han	16,214 (97.1)	1,956 (96.8)	0.593	17,820 (97.1)	350 (96.7)	0.800
Others	489 (2.9)	64 (3.2)		541 (2.9)	12 (3.3)	
**Registered permanent residence**, ***n*** **(%)**						
Rural	9,061 (54.2)	1,056 (52.3)	0.098	9,954 (54.2)	163 (45)	< 0.001
Urben	7,642 (45.8)	964 (47.7)		8,407 (45.8)	199 (55)	
**Siblings**, ***n*** **(%)**						
No	4,897 (29.3)	632 (31.3)	0.071	5,411 (29.5)	118 (32.6)	0.217
Yes	11,806 (70.7)	1,388 (68.7)		12,950 (70.5)	244 (67.4)	
**Maternal educational attainment**, ***n*** **(%)**						
Middle school or under	4,999 (29.9)	604 (29.9)	0.195	5,499 (29.9)	104 (28.7)	0.063
High school	5,784 (34.6)	653 (32.3)		6,322 (34.4)	115 (31.8)	
College or above	5,920 (35.4)	763 (37.8)		6,540 (35.6)	143 (39.5)	
**Parental educational attainment**, ***n*** **(%)**						
Middle school or under	3,451 (20.7)	446 (22.1)	0.037	3,814 (20.8)	83 (22.9)	< 0.001
High school	5,730 (34.3)	605 (30)		6,238 (34)	97 (26.8)	
College or above	7,522 (45)	969 (48)		8,309 (45.3)	182 (50.3)	
**BMI**, ***n*** **(%)**						
Normal weight	13,125 (78.8)	1,534 (76.3)	0.009	14,375 (78.6)	284 (78.5)	1.000
Overweight or obesity	3,521 (21.2)	477 (23.7)		3,920 (21.4)	78 (21.5)	
**Smoking**, ***n*** **(%)**						
No	14,776 (88.5)	1,685 (83.4)	< 0.001	16,177 (88.1)	284 (78.5)	< 0.001
Yes	1,927 (11.5)	335 (16.6)		2,184 (11.9)	78 (21.5)	
**Drinking**, ***n*** **(%)**						
No	13,656 (81.8)	1,500 (74.3)	< 0.001	14,917 (81.2)	239 (66)	< 0.001
Yes	3,047 (18.2)	520 (25.7)		3,444 (18.8)	123 (34)	
**Sleep disorders**, ***n*** **(%)**						
No	14,319 (85.7)	1,339 (66.3)	< 0.001	15,476 (84.3)	182 (50.3)	< 0.001
Yes	2,384 (14.3)	681 (33.7)		2,885 (15.7)	180 (49.7)	
**Depression symptoms**, ***n*** **(%)**						
No	15,895 (95.2)	1,579 (78.2)	< 0.001	17,233 (93.9)	241 (66.6)	< 0.001
Yes	808 (4.8)	441 (21.8)		1,128 (6.1)	121 (33.4)	
Social support score, mean (SD)	67.9 (14.9)	59.9 (15.6)	< 0.001	67.3 (15.1)	56.9 (15.7)	< 0.001
MPATS- salience, mean (SD)	15.8 (5.3)	18 (5.7)	< 0.001	16 (5.3)	18.8 (5.8)	< 0.001
MPATS-withdrawal symptoms, mean (SD)	8.5 (3.3)	10.2 (3.8)	< 0.001	8.6 (3.3)	10.6 (4)	< 0.001
MPATS-social comfort, mean (SD)	7.4 (2.9)	8.8 (3.2)	< 0.001	7.5 (3)	9.4 (3.2)	< 0.001
MPATS-mood changes, mean (SD)	7 (2.8)	8.3 (3)	< 0.001	7.1 (2.8)	8.9 (2.9)	< 0.001
MPATS score, mean (SD)	38.8 (12.7)	45.3 (14)	< 0.001	39.3 (13)	47.7 (14.2)	< 0.001
**Mobile phone addiction**, ***n*** **(%)**						
No	12,134 (72.6)	1,036 (51.3)	< 0.001	13,003 (70.8)	167 (46.1)	< 0.001
Yes	4,569 (27.4)	984 (48.7)		5,358 (29.2)	195 (53.9)	

The associations of mobile phone addiction with suicide ideation and suicide attempt are shown in Table 2. Among participants with mobile phone addiction, the rates of suicide ideation and suicide attempt were 17.7% and 3.5%, respectively. Mobile phone addiction was positively correlated with an increased risk of suicide ideation and suicide attempt. After adjusting for gender, grade, and race (Table 2, Model 2), mobile phone addiction was significantly related to increased odds of suicide ideation (OR, 2.51; 95% CI, 2.28–2.75) and suicide attempt (OR, 2.81; 95% CI, 2.28–3.47). Further adjusting for siblings, registered permanent residence, parental educational attainment, and health-related characteristics (smoking, drinking, BMI) (Table 2, Model 3), the ORs were reduced slightly. In fully adjusted model further adjusted for depressive symptoms, social support, and sleep disturbances (Table 2, Model 4), mobile phone addiction remained positively associated with suicidal ideation (OR, 1.70; 95% CI, 1.53–1.88) and suicide attempt (OR, 1.48; 95% CI, 1.18–1.86), despite a substantial decrease in ORs. Each five points increase in MPATS score was significantly associated with the higher odds of suicide ideation (OR, 1.12; 95% CI, 1.09–1.14) and suicide attempt (OR, 1.11; 95% CI, 1.06–1.15). Similar significant results were observed for each dimension of MPATS (Table 2).

**Table 2:** Associations of mobile phone addiction with suicide ideation and suicide attempt.

**Models**	**OR (95% CI)**	
	**Suicide ideation**	**Suicide attempt**
**Mobile phone addiction**		
Model 1	2.52 (2.30–2.77)	2.83 (2.30–3.50)
Model 2	2.51 (2.28–2.75)	2.81 (2.28–3.47)
Model 3	2.44 (2.22–2.68)	2.62 (2.13–3.24)
Model 4	1.70 (1.53–1.88)	1.48 (1.18–1.86)
**MPATS score, per 5-unit increases**		
Model 1	1.21 (1.19–1.23)	1.27 (1.22–1.32)
Model 2	1.21 (1.19–1.23)	1.27(1.22–1.32)
Model 3	1.20 (1.18–1.22)	1.25 (1.20–1.30)
Model 4	1.12 (1.09–1.14)	1.11 (1.06–1.15)
**Withdrawal symptoms, per unit increases**		
Model 1	1.08 (1.07–1.09)	1.10 (1.08–1.13)
Model 2	1.08 (1.07–1.09)	1.10 (1.08–1.13)
Model 3	1.08 (1.07–1.08)	1.09 (1.07–1.11)
Model 4	1.04 (1.03–1.05)	1.04 (1.01–1.06)
**Salience, per unit increases**		
Model 1	1.15 (1.13–1.16)	1.17 (1.13–1.20)
Model 2	1.15 (1.13–1.16)	1.17 (1.13–1.20)
Model 3	1.14 (1.13–1.16)	1.16 (1.12–1.19)
Model 4	1.08 (1.07–1.10)	1.06 (1.02–1.09)
**Social comfort, per unit increases**		
Model 1	1.16 (1.15–1.18)	1.21 (1.17–1.25)
Model 2	1.16 (1.15–1.18)	1.21 (1.17–1.25)
Model 3	1.16 (1.14–1.18)	1.20 (1.16–1.24)
Model 4	1.10 (1.08–1.12)	1.10 (1.06–1.14)
**Mood changes, per unit increases**		
Model 1	1.17 (1.15–1.19)	1.24 (1.19–1.28)
Model 2	1.17 (1.15–1.19)	1.23 (1.19–1.28)
Model 3	1.16 (1.14–1.18)	1.22 (1.17–1.26)
Model 4	1.09 (1.07–1.11)	1.10 (1.06–1.14)

Model 1, crude model.

Model 2, adjustment for gender, grade, and race.

Model 3, adjustment for gender, grade, race, registered permanent residence, siblings, maternal educational attainment, paternal educational attainment, BMI, smoking, and drinking.

Model 4, adjustment for gender, grade, race, registered permanent residence, siblings, maternal educational attainment, paternal educational attainment, BMI, smoking, drinking, sleep disorders, depressive symptoms, and social support.

**Figure 1:**
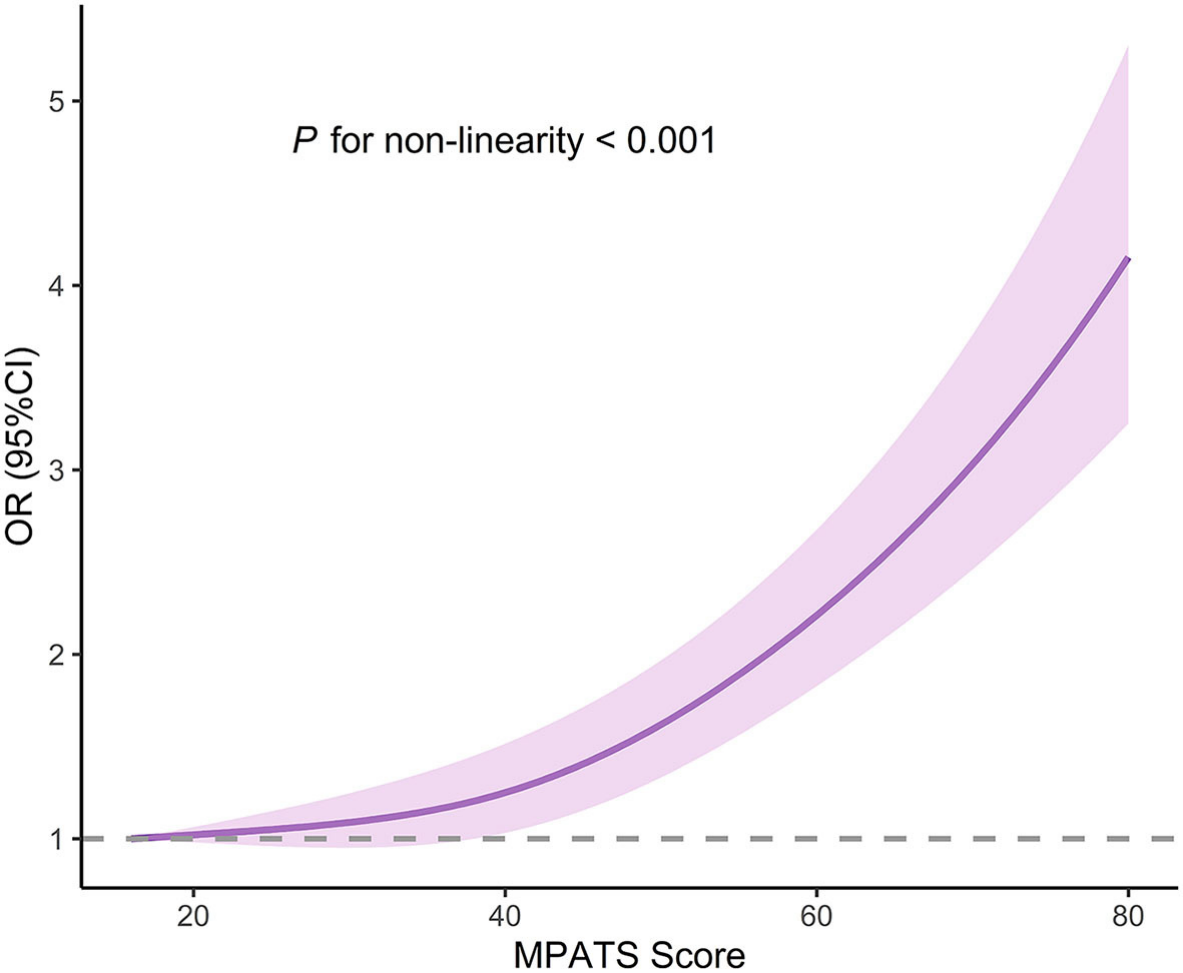
Restricted cubic splines regression analysis of MPATS score with suicide ideation risk. OR, odds ratio; CI, confidence interval. The analysis was adjusted for gender, grade, race, registered permanent residence, siblings, parental educational attainment, BMI, smoking, drinking, sleep disorder, depressive symptoms, and social support.

**Figure 2:**
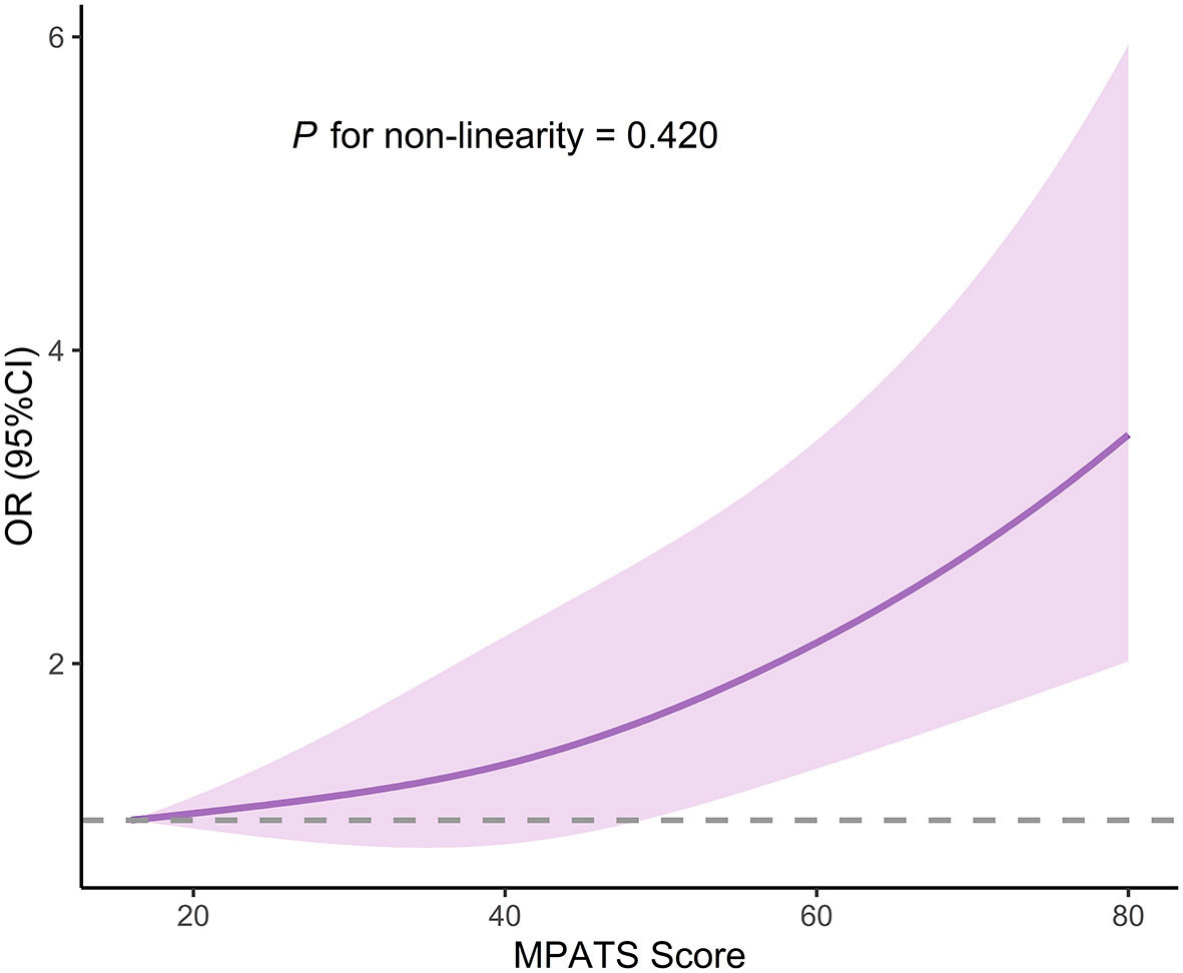
Restricted cubic splines regression analysis of MPATS score with suicide attempt risk. OR, odds ratio; CI, confidence interval. The analysis was adjusted for gender, grade, race, registered permanent residence, siblings, parental educational attainment, BMI, smoking, drinking, sleep disorder, depressive symptoms, and social support.

Table 3 displays the findings of the gender-stratified analysis. In male participants, mobile phone addiction was associated with suicide ideation (OR, 1.70; 95% CI, 1.42–2.03), but was not statistically significant in relation to suicide attempt (OR, 1.29; 95% CI, 0.86–1.95). For female participants, mobile phone addiction was found positive correlated with risk of suicide ideation (OR, 1.69; 95% CI, 1.49–1.91) and suicide attempt (OR, 1.59; 95% CI, 1.21–2.1). The current study found no significant gender-specific differences in the relationships of mobile phone addiction with suicide ideation and suicide attempt were found in the present study (*P for interaction* > *0.05*). Moreover, the restricted cubic splines regression revealed a non-linear association between MPATS score and suicide ideation (*P for non-linearity* < 0.001; Figure 1), whereas a monotonically increasing risk of suicide attempt was found to be associated with increasing MPATS score (*P for non-linearity* = 0.420; Figure 2).

**Table 3:** Associations of mobile phone addiction with suicide ideation and suicide attempt, stratified by gender.

**Mobile phone addiction**	**Suicide ideation**		***P* for interaction**	**Suicide attempt**		***P* for interaction**
	**Male**	**Female**		**Male**	**Female**	
Mobile phone addiction	1.70 (1.42–2.03)	1.69 (1.49–1.91)	0.841	1.29 (0.86–1.95)	1.59 (1.21–2.10)	0.441
MPATS score, per 5-unit increases	1.11 (1.07–1.14)	1.12 (1.09–1.15)	0.563	1.08 (1.00–1.16)	1.12 (1.06–1.18)	0.485
Salience, per unit increases	1.04 (1.02–1.05)	1.04 (1.03–1.06)	0.472	1.02 (0.99–1.06)	1.04 (1.02–1.07)	0.524
Withdrawal symptoms, per unit increases	1.08 (1.05–1.11)	1.08 (1.06–1.10)	0.976	1.05 (0.99–1.11)	1.06 (1.02–1.10)	0.768
Social comfort, per unit increases	1.11 (1.08–1.14)	1.09 (1.07–1.12)	0.512	1.09 (1.02–1.16)	1.11 (1.06–1.16)	0.562
Mood changes, per unit increases	1.09 (1.06–1.12)	1.09 (1.07–1.11)	0.910	1.09 (1.02–1.17)	1.11 (1.06–1.16)	0.801

Adjusted for gender, grade, race, registered permanent residence, siblings, maternal educational attainment, paternal educational attainment, BMI, smoking, drinking, sleep disorders, depressive symptoms, and social support.

## 4 Discussion

The present study was conducted to investigate the associations of mobile phone addiction with suicide ideation and suicide attempt. Compared with college students without mobile phone addiction, those who engage in mobile phone addiction had significant increased odds of suicide ideation and suicide attempt.

### 4.1 Comparison with other studies

Mobile phone ownership has been increasing over the past decade, and the problematic mobile phone use has raised concerns about mobile phone addiction. A review across 24 countries identified that the prevalence of mobile phone addiction is increasing around the world from 2014 to 2020, and differs considerably by country (13). Prior studies (18, 36–38) have reported varied prevalence of mobile phone addiction from 14% to 48% among college students across different countries. In China, several studies have shown a comparatively high prevalence of mobile phone addiction among youths (13, 39). The present study conducted among six universities in Shannxi Province of China showed a 29.7% prevalence of mobile phone addiction, which was comparable to the majority of previous studies (39–42). For example, a survey from Anhui Province of China (43), reported a 29.8% rate of mobile phone addiction among college students. Geng et al. (44) also presented a 23.5% prevalence of mobile phone addiction in medical college students in China. However, it is lower than the study from Lei et al. (16), which showed a 40.6% prevalence of mobile phone addiction among 574 medical students in a public medical university. The variation in the prevalence of mobile phone addiction among different studies could be related to the study population, the assessment tool, and the location of the survey. In this study, the population was comprised of students from six universities, covering different majors and grades, and it may have overcome the limitations of the representative sample population in prior studies performed at only one university or only from specific majors. As relevant research is limited at present, there is a need for further research in a nationally representative population.

Given the comparatively high prevalence of mobile phone addiction among students, it is necessary to know about the impact of mobile phone addiction on suicide of current college students, especially among iGens who grow up with the surroundings of mobile phones. To our best knowledge, only five studies have examined the associations of mobile phone addiction with suicide, but conclusions are not entirely consistent. Four studies indicated a positive association between mobile phone addiction and suicide ideation or suicide attempt (25–28), while one study found no relationship of mobile phone addiction and suicide (29). Some other studies suggest that the length of mobile phone use is linked to suicide. For example, Zhang et al. (45) found that longer duration of mobile phone use predicted suicide behaviors in Chinese adolescents. Huang et al. (29) also reported that students with suicide ideation may have longer mobile phone use time. The findings in our survey were consistent with majority of previous studies, which suggested that students with mobile phone addiction had significant higher prevalence of suicide, extending the limited available evidence on the association between mobile phone addiction and increased odds of suicide in iGens. Thus, our study is important for considering the significant role of mobile phone addiction when developing suicide prevention programs for college students.

To our knowledge, no study has examined gender differences in the association between mobile phone addiction and suicide. While most previous studies have suggested that women may demonstrate distinct patterns when confronting mental health issues, this study did not uncover substantial gender disparities in the prevalence of suicide ideation and suicide attempt. Furthermore, there was no significant gender-smartphone interaction on the likelihood of suicide ideation and suicide attempt identified in this study. These findings indirectly reflect the importance of giving equal consideration to different genders in future interventions related to mobile phone usage. Moreover, our study may be the first study to evaluate the dose-response relationship of mobile phone addiction with suicide ideation and suicide attempt. We found a non-linear association between mobile phone addiction score and suicide ideation, and a monotonically increasing relationship between mobile phone addiction score and suicide attempt. However, because the related study is still limited so far, further longitudinal investigations or intervention studies on the association between mobile phone addiction and suicide ideation/attempt are warranted.

### 4.2 Possible explanations of the association

Suicide is a complex social phenomenon, which is generated by the interaction of biological, psychological, and social perspectives factors. The exact mechanism underlying its association with mobile phone addiction is complex and unclear. The Integrated Motivation-Volition Model of Suicidal Behavior (IMV) proposed that suicide develops through pre-motivational phase (the transitions from the defeat/humiliation stage to entrapment), the motivational phase (from entrapment to ideation/intent), and the volitional phase (from ideation/intent to behavior) (22). Whether an individual will act on their suicidal ideation/intent is determined by a range of factors, labeled volitional moderators. Impulsivity, having the capability to attempt suicide, knowing others who engage in suicide, and having access to the means of suicide are examples of volitional moderators. According to IMV, on the one hand, students who are addicted to mobile phones may be more likely to access suicide-related information and learn about suicide tactics, and thus more likely to imitate and commit suicide. On the other hand, mobile phone addiction can lead to depersonalization, affecting the normal social functioning of adolescents and reducing emotions such as alertness and fear. Ultimately, it tends to produce impulse inhibition disorder, which can increase the risk of suicide. Furthermore, previous studies have identified depression and sleep as mediating variables between mobile phone addiction and suicide. For example, Zhang et al. (45) found the link between mobile phone use and suicide was partially mediated by depressive symptoms. In the present study, we also found that students with suicide ideation or attempt had more depression and bad sleep quality. Nevertheless, the associations of mobile phone addiction with suicide ideation and suicide attempt were still significant after additional adjustments for depression and sleep quality, indicating the independent effect of mobile phone addiction on the risk of suicide.

Internet addiction and mobile phone addiction are both technology addiction, which refers to behavior addictions that associated with the excessive and uncontrolled use of technology, with similar characteristics and formation mechanisms (46). Previous research on internet addiction may provide evidence of the link between mobile phone addiction and suicide. First, it is widely accepted that the hypothalamic-pituitary-adrenal (HPA) axis dysregulation is the important pathophysiology mechanism for suicide. 5-HT is a key neurotransmitter of HPA (47), and its reduction is believed to be associated with suicide. Cerniglia et al. (48) have shown that the decrease of 5-HT is also related to the dysfunction of the prefrontal cortex caused by internet addiction. Hence, 5-HT may also be responsible for the association between mobile phone addiction and suicide. Second, changes in brain structure and function may be the neurobiological mechanisms between mobile phone addiction and suicide. Research have showed that people with internet addiction are prone to abnormal changes in the structure as well as function of the gray and white matter in the prefrontal lobe of the brain (49, 50). In addition, several studies have highlighted that the prefrontal lobe also plays an important role in suicide (51, 52), which may indirectly confirm the results of this study. However, to understand the detailed mechanisms underlying the relationship of mobile phone addiction with suicide ideation and suicide attempt, more cohort or intervention studies are needed.

### 4.3 Limitations

This study has some limitations. First, the collection of suicide ideation, suicide attempt, and mobile phone addiction information through a self-report questionnaire may result in recall bias. Second, although most of the available important covariates were included in this study, some residual or unmeasured confounding parameters may had an impact on the results. Last, the cross-sectional study design limits the causal inferences of this study.

## 5 Conclusions

The findings of our study suggest that mobile phone addiction is associated with increased risk of suicide ideation and suicide attempt. Mobile phone addiction should thus be considered in intervention programs for the aim of reducing the rate of suicide, especially among iGens.

## Data availability statement

The raw data supporting the conclusions of this article will be made available by the authors, without undue reservation.

## Ethics statement

The studies involving humans were approved by the Second Affiliated Hospital of Xi'an Jiaotong University. The studies were conducted in accordance with the local legislation and institutional requirements. The participants provided their written informed consent to participate in this study. Written informed consent was obtained from the individual(s), and minor(s)' legal guardian/next of kin, for the publication of any potentially identifiable images or data included in this article.

## Author contributions

WW: Data curation, Formal analysis, Investigation, Project administration, Writing – original draft, Writing – review & editing. MW: Writing – original draft, Writing – review & editing. ZZ: Methodology, Project administration, Supervision, Validation, Writing – review & editing. LM: Writing-review & editing, Methodology, Supervision, Validation, and Visualization. LZ: Funding acquisition, Investigation, Methodology, Project administration, Resources, Supervision, Validation, Visualization, Writing – review & editing, Conceptualization. HL: Methodology, Supervision, Writing – review & editing, Conceptualization, Project administration, Validation, Visualization.
